# Hybrid Congenital Pulmonary Malformation Revealed by Chronic Hemoptysis in a Six-Year-Old Girl: A Case Report

**DOI:** 10.7759/cureus.108001

**Published:** 2026-04-29

**Authors:** Younes Abdourabbih, Adnane Amelouagh, Hajar Ouazzani, Ismail Chaouche, Amal Akammar, Nizar El Bouardi, Badreddine Alami, Y. Lamrani, Mustapha Maaroufi, Meryem Boubbou

**Affiliations:** 1 Radiology, Centre Hospitalier Universitaire Hassan II, Sidi Mohamed Ben Abdellah University, Fes, MAR; 2 Radiology, Centre Hospitalier Universitaire Hassan II, Sidi Mohamed Ben Abdellah University, Fez, MAR; 3 Maternal and Pediatric Radiology, Centre Hospitalier Universitaire Hassan II, Sidi Mohamed Ben Abdellah University, Fez, MAR

**Keywords:** bronchopulmonary sequestration, child, congenital lung malformation, congenital pulmonary airway malformation, ct angiography, hemoptysis, hybrid lesion

## Abstract

Congenital lung malformations (CLMs) are developmental conditions characterized by overlapping clinical and imaging features, with hybrid lesions that combine congenital pulmonary airway malformation (CPAM) and bronchopulmonary sequestration (BPS) becoming increasingly recognized by multidetector CT (MDCT). We present the case of a six-year-old girl with a one-year history of recurrent low-volume hemoptysis. Her chest radiograph indicated a subtle opacity in the right basal area. Thoracic CT angiography revealed parenchymal consolidation in the right lower lobe, characterized by an air bronchogram that did not connect with the tracheobronchial tree and was accompanied by a well-defined thin-walled cystic lesion. This lesion was vascularized by a systemic artery originating from the anterior descending thoracic aorta and drained anomalously into the left atrium, a finding consistent with a hybrid congenital pulmonary airway malformation and sequestration. This case emphasizes the critical importance of MDCT angiography in accurately delineating parenchymal and cystic components, detecting blind-ending noncommunicating bronchi, and charting anomalous arterial supply and venous drainage, consequently differentiating hybrid CLMs from other congenital or acquired disorders and offering a vital vascular framework for surgical planning. In pediatric patients exhibiting unexplained hemoptysis or chronic focal pulmonary opacities, early CT angiography is therefore recommended to detect and accurately characterize CLMs and guide multidisciplinary management.

## Introduction

Congenital lung malformations (CLMs) comprise a spectrum of developmental anomalies, including congenital pulmonary airway malformation (CPAM) and bronchopulmonary sequestration (BPS), with overlapping histologic and radiologic features that may coexist in hybrid lesions combining dysplastic airway/cystic components and anomalous systemic vascularization [1-3]. Clinical presentation is variable and may include prenatal mediastinal mass effect, recurrent pulmonary infections, chronic cough, hemoptysis, pneumothorax, or incidental detection on imaging. Prenatal detection relies primarily on ultrasound with occasional identification of systemic feeders on color Doppler; fetal MRI provides a complementary assessment of lesion extent, mediastinal effects, and lung development when ultrasound is equivocal [3]. Postnatally, cross‑sectional angiographic imaging-multidetector CT angiography (MDCT‑CTA) or contrast‑enhanced MR angiography plays a central role in delineating parenchymal morphology, bronchial communication, and the number, origin, and course of anomalous systemic arteries and venous drainage, which critically influence management [4,5]. Increasing recognition of hybrid CPAM-BPS lesions in the literature underscores the need for systematic vascular mapping and multidisciplinary evaluation to guide individualized therapy [6-8].

## Case presentation

A six‑year‑old girl with no relevant past medical or family history presented for evaluation of intermittent hemoptysis over approximately one year. The episodes consisted of blood‑streaked sputum and small‑volume hemoptysis, occurring several times per month, without massive bleeding, dyspnea, or hemodynamic compromise. There was no associated fever, chronic productive cough, weight loss, night sweats, or prior history of pneumonia, aspiration, or tuberculosis exposure. No history of coagulopathy, cardiac disease, or other congenital anomalies was reported.

On physical examination, the patient was in good general condition. Vital signs were within normal limits, and oxygen saturation was 98% on room air. Chest auscultation revealed faint fine crackles over the right lung base; the remainder of the respiratory and systemic examination was unremarkable.

Initial laboratory testing (Table 1) showed hemoglobin 12.9 g/dL, leukocytosis (WBC 16.33 × 10^3/µL) with eosinophilia (11.9%), and platelets 343 × 10^3/µL. C‑reactive protein was 1.0 mg/L (reference range: 0-5 mg/L). Coagulation studies were within reference ranges (activated partial thromboplastin time (aPTT) 31.9 seconds (27.6-38.4), prothrombin time (PT) 100%, international normalised ratio (INR) 1.00); coagulopathy was, therefore, excluded based on both history and normal coagulation tests. The leukocytosis and eosinophilia were evaluated clinically and considered most likely reactive in the absence of elevated CRP or other evidence of acute bacterial infection.

**Table 1:** Laboratory investigations at presentation abs: absolute

Test	Result	Reference range
Red blood cell (RBC)	5.29 ×10^6/µL	3.80–5.50 ×10^6/µL
Hemoglobin (Hgb)	12.90 g/dL	11.5–15.5 g/dL
Hematocrit (Hct)	41.5%	35–45%
Mean corpuscular volume (MCV)	78.4 fL	76–90 fL
Mean corpuscular hemoglobin (MCH)	24.4 pg	24–30 pg
Mean corpuscular hemoglobin concentration (MCHC)	31.1 g/dL	31–36 g/dL
Leucocytes (WBC)	16.33 ×10^3/µL	5.0–14.5 ×10^3/µL
Neutrophils (abs)	9.56 ×10^3/µL	1.5–8.5 ×10^3/µL
Neutrophils (%)	58.6%	30–60%
Eosinophils (abs)	1.95 ×10^3/µL	0.05–0.5 ×10^3/µL
Eosinophils (%)	11.9%	0–6%
Basophils (abs)	0.15 ×10^3/µL	0–0.1 ×10^3/µL
Basophils (%)	0.9%	0–1%
Lymphocytes (abs)	3.68 ×10^3/µL	1.5–7.0 ×10^3/µL
Lymphocytes (%)	22.5%	20–50%
Monocytes (abs)	0.99 ×10^3/µL	0.1–1.0 ×10^3/µL
Monocytes (%)	6.1%	2–8%
Platelets	343 ×10^3/µL	150–400 ×10^3/µL
C‑reactive protein (CRP)	1.0 mg/L	0.0–5.0 mg/L
Sodium (Na)	136 mmol/L	135–145 mmol/L
Potassium (K)	3.7 mEq/L	3.5–4.5 mEq/L
Urea	0.11 g/L	0.17–0.43 g/L
Creatinine	3.0 mg/L	4.0–14.0 mg/L
Activated partial thromboplastin time (aPTT)	31.9 seconds	27.6–38.4 seconds
Prothrombin time (PT)	100%	Laboratory reference
International normalized ratio (INR)	1.00	Laboratory reference

**Figure 1:**
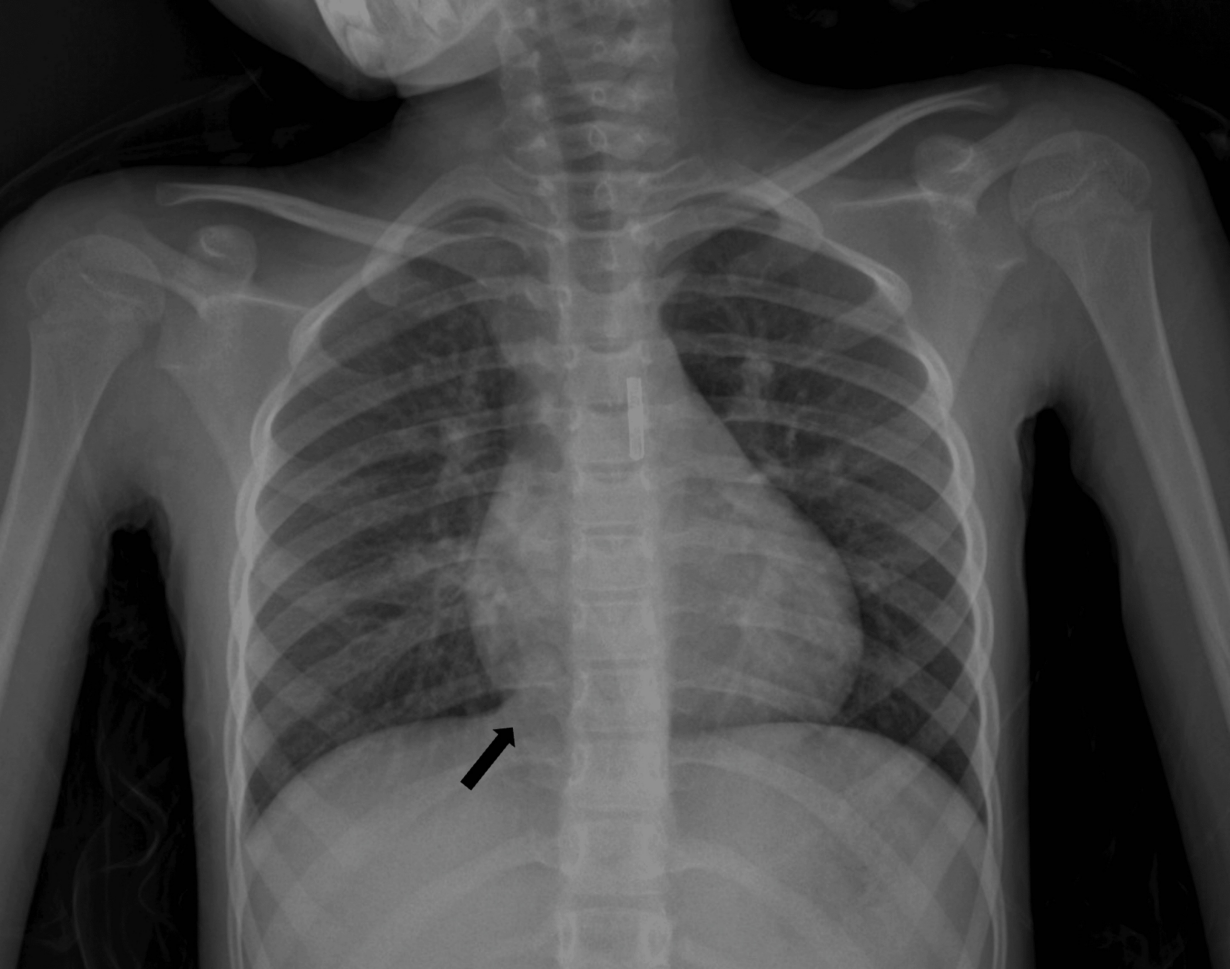
Posteroanterior chest radiograph Right paracardiac consolidation (black arrow) along the lateral aspect of the right heart border without obliteration of the cardiac silhouette is noted.

Posteroanterior chest radiography shows a right paracardiac area of consolidation along the lateral aspect of the right heart border that does not obscure the cardiac contours (Figure 1). Given the persistence of hemoptysis and the inconclusive radiographic findings, a contrast‑enhanced thoracic CT with angiographic acquisition was performed for further evaluation.

Imaging findings 

**Figure 2:**
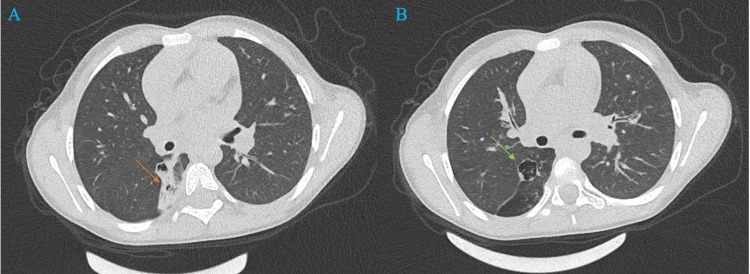
Axial chest CT (lung window) A: Right lower‑lobe parenchymal consolidation with air bronchograms (orange arrow); B: Thin‑walled, air‑filled cystic lesion in the posterior basal segment of the right lower lobe (green arrow).

Thoracic CT angiography demonstrated a well‑demarcated parenchymal consolidation in the right lower lobe, respecting lobar anatomy, with homogeneous soft‑tissue attenuation. An air bronchogram coursed through the consolidated area; on multiplanar reconstructions, this bronchus appeared blind-ending and did not communicate with the segmental or lobar bronchi of the right lower lobe, suggesting an aberrant or isolated bronchial segment (Figure 2A).

Immediately superior to the consolidated focus, there was a single, well‑circumscribed, thin‑walled cystic lesion measuring 35 × 28 × 30 mm, predominantly air‑filled with thin intracystic septations and without mural nodules or wall thickening (Figure 2B). No surrounding ground‑glass opacity, fat stranding, or air‑fluid level suggested acute infection.

**Figure 3:**
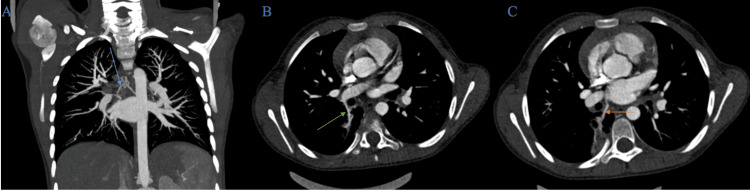
Chest CT angiography (mediastinal window) in coronal plane with maximum intensity projection (A) and axial plane (B, C) A systemic arterial feeder arising from the anterior aspect of the descending thoracic aorta (blue arrow), coursing toward the right lower lobe and entering the consolidation, is seen. A branch coming from the right inferior pulmonary vein (green arrow) and an anomalous venous drainage channel draining directly into the left atrium (orange arrow) is visualized.

Vascular assessment demonstrated a systemic arterial feeder arising from the anterior aspect of the descending thoracic aorta. This vessel coursed anteriorly and laterally toward the right lower lobe and entered the area of consolidation (Figure 3A), providing the dominant arterial supply. We also identified a branch arising from the lower lobe pulmonary artery (Figure 3B) and an anomalous venous drainage pathway with a vein draining directly into the left atrium (Figure 3C), separate from the normal right inferior pulmonary vein.

The remaining lung parenchyma was unremarkable, with no additional cystic, solid, or consolidative lesions. The trachea and central bronchi were normal in caliber and course, apart from the blind‑ending bronchus within the lesion. The mediastinum and pleural spaces were normal, with no lymphadenopathy or effusion.

**Figure 4:**
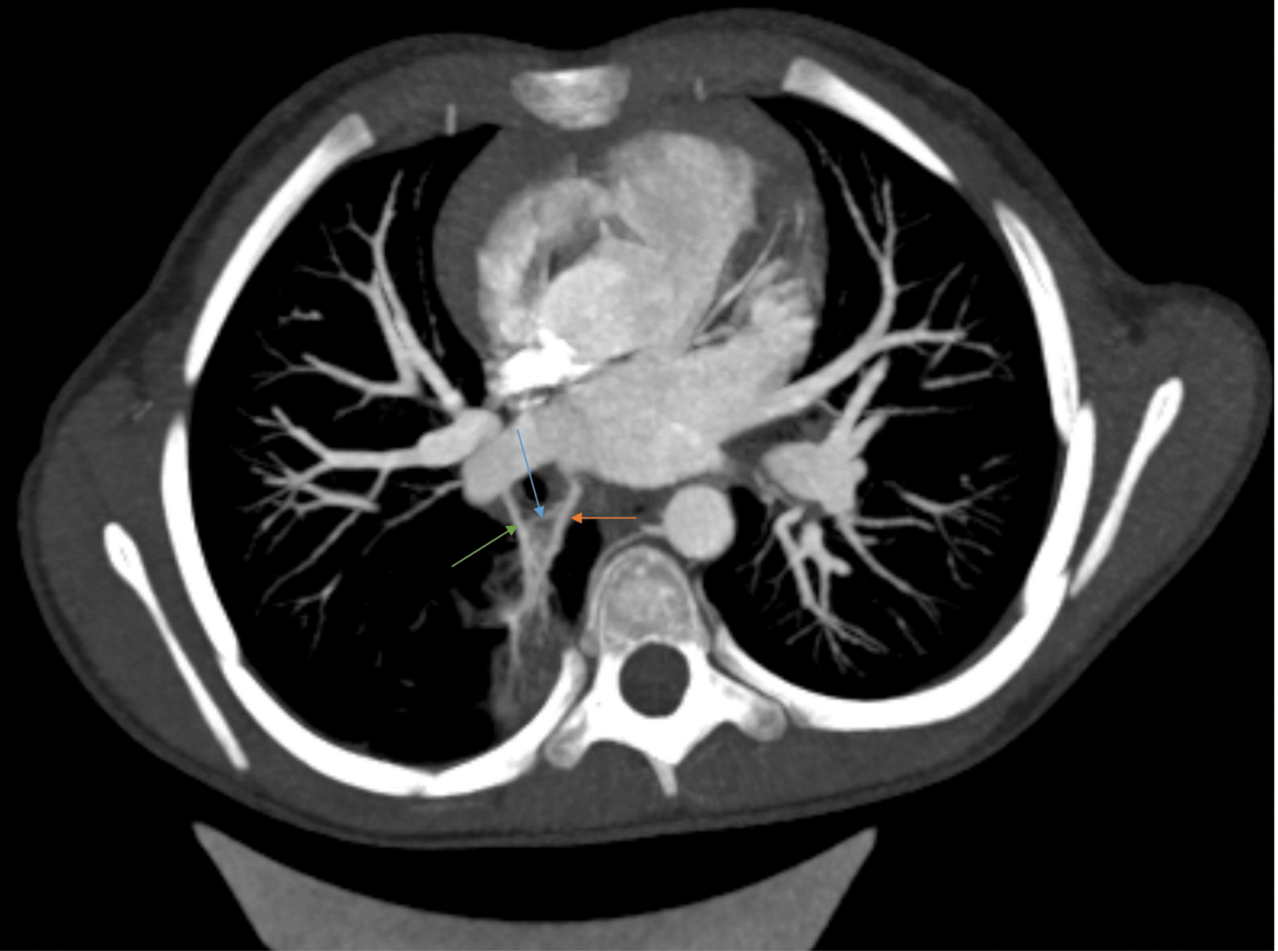
Chest CT angiography (mediastinal window) in axial plane with maximum intensity projection All three branches together: blue arrow, arterial supply from the descending aorta; green arrow, branch coming from the right inferior pulmonary vein; orange arrow, anomalous venous drainage channel draining directly into the left atrium.

The combination of a localized segment of abnormal parenchyma harboring a blind‑ending, non‑communicating bronchus, a contiguous, well‑defined, thin‑walled cystic component, and an aberrant systemic arterial supply from the descending thoracic aorta with anomalous venous drainage to the left atrium (Figure 4) was consistent with a hybrid congenital pulmonary malformation incorporating features of CPAM and intralobar BPS.

Management and outcome 

The case was discussed in a multidisciplinary meeting involving pediatric pulmonology, pediatric surgery, and radiology. Given the presence of symptoms (recurrent hemoptysis), the abnormal systemic arterial supply, and the potential risk of future complications, surgical resection of the affected right lower lobe was recommended. After counseling, the parents declined operative intervention. The patient was therefore managed conservatively with a structured follow‑up plan (scheduled clinical assessments, clear escalation thresholds for increasing or large‑volume hemoptysis or respiratory compromise, interval vascular imaging to monitor feeder anatomy, and prompt treatment of infections).

## Discussion

Hybrid pulmonary malformations, lesions showing both CPAM‑type cystic or airway‑derived dysplasia and anomalous systemic arterial feeders typical of BPS, pose diagnostic and therapeutic challenges because vascular anatomy alters natural history and operative risk [1,2]. Reported presentations vary (prenatal mass effect, recurrent infections, hemoptysis, pneumothorax, and incidental detection), and vascular anatomy is heterogeneous (thoracic or abdominal aortic feeders, mixed arterial supply, and pulmonary versus systemic venous drainage), making high‑resolution angiographic mapping mandatory before intervention [6-9].

MDCT‑CTA provides submillimetric depiction of cystic versus solid components, bronchial communication, and the precise origin, number, and caliber of feeders and venous outflow, data that directly affect decisions between observation, embolization, segmentectomy, or lobectomy and reduce the risk of intraoperative hemorrhage [3-5]. In this case, MDCT‑CTA demonstrated a blind‑ending noncommunicating bronchus and a contiguous thin‑walled cystic component consistent with CPAM‑type dysplasia, together with a dominant systemic feeder from the descending thoracic aorta, an additional pulmonary arterial branch (dual arterial supply), and anomalous venous drainage to the left atrium, a combination supporting hybrid classification. Dual or multiple arterial feeders and anomalous venous outflow increase theoretical intraoperative hemorrhage risk and would influence consideration of preoperative embolization and surgical approach.

Differential diagnoses for focal cystic or consolidated lung lesions in children include infected cavitary lesions, bronchogenic cysts, congenital lobar emphysema, pulmonary arteriovenous malformations, and neoplasms; distinguishing features include the presence or absence of systemic arterial supply, bronchial communication, wall characteristics, and inflammatory markers. MDCT‑CTA is usually decisive in differentiating these entities.

Therapeutic choice should integrate clinical symptoms, lesion behavior, and imaging anatomy. Symptomatic lesions or those with recurrent infection commonly prompt resection; endovascular embolization may reduce perioperative bleeding or act as temporizing therapy, but embolization alone may leave dysplastic lung tissue in situ and not eliminate infection or other long-term risks [8,10,11]. When families decline surgery, as in this case, structured conservative surveillance with clear escalation triggers is essential. This entails scheduled clinical review with clear escalation thresholds (large‑volume or increasing hemoptysis, respiratory compromise, fever, or focal neurologic signs); interval vascular imaging to monitor feeder anatomy and lesion stability; prompt treatment of infections; and timely reconsideration of embolization or delayed resection if symptoms progress [3,5]. Case reports of hemoptysis and chronic cough highlight that even small or previously asymptomatic hybrid lesions may present later in childhood or adulthood and that CT angiography is decisive in these scenarios [12,13,14].

## Conclusions

Hybrid congenital pulmonary malformations are rare entities that lie at the intersection of CPAM and BPS within the spectrum of CLMs. In this six‑year‑old girl with chronic low‑volume hemoptysis, thoracic CT angiography was decisive in establishing the diagnosis by demonstrating a blind‑ending, non‑communicating bronchus, a contiguous thin‑walled cystic lesion, and aberrant systemic arterial supply with anomalous venous drainage into the left atrium. CT angiography not only clarifies the nature of the lesion but also narrows the differential diagnosis among CLMs. Early use of CT angiography should be considered in pediatric patients with unexplained hemoptysis or persistent focal pulmonary opacities to detect and accurately characterize CLMs, including rare hybrid forms, and to guide timely multidisciplinary management.
